# Extracellular flux analyses reveal differences in mitochondrial PBMC metabolism between high-fit and low-fit females

**DOI:** 10.1152/ajpendo.00365.2021

**Published:** 2022-01-10

**Authors:** Joëlle J. E. Janssen, Bart Lagerwaard, Mojtaba Porbahaie, Arie G. Nieuwenhuizen, Huub F. J. Savelkoul, R. J. Joost van Neerven, Jaap Keijer, Vincent C. J. de Boer

**Affiliations:** ^1^Human and Animal Physiology, Wageningen University and Research, Wageningen, The Netherlands; ^2^Cell Biology and Immunology, Wageningen University and Research, Wageningen, The Netherlands; ^3^TI Food and Nutrition, Wageningen, The Netherlands

**Keywords:** aerobic fitness level, biomarker, lifestyle, oxygen consumption rate, PBMC metabolism

## Abstract

Analyzing metabolism of peripheral blood mononuclear cells (PBMCs) can possibly serve as a cellular metabolic read-out for lifestyle factors and lifestyle interventions. However, the impact of PBMC composition on PBMC metabolism is not yet clear, neither is the differential impact of a longer-term lifestyle factor versus a short-term lifestyle intervention. We investigated the effect of aerobic fitness level and a recent exercise bout on PBMC metabolism in females. PBMCs from 31 young female adults divided into a high-fit (V̇o_2peak_ ≥ 47 mL/kg/min, *n* = 15) and low-fit (V̇o_2peak_ ≤ 37 mL/kg/min, *n* = 16) groups were isolated at baseline and overnight after a single bout of exercise (60 min, 70% V̇o_2peak_). Oxygen consumption rate (OCR) and glycolytic rate (GR) were measured using extracellular flux (XF) assays and PBMC subsets were characterized using fluorescence-activated cell sorting (FACS). Basal OCR, FCCP-induced OCR, spare respiratory capacity, ATP-linked OCR, and proton leak were significantly higher in high-fit than in low-fit females (all *P* < 0.01), whereas no significant differences in glycolytic rate (GR) were found (all *P* > 0.05). A recent exercise bout did not significantly affect GR or OCR parameters (all *P* > 0.05). The overall PBMC composition was similar between high-fit and low-fit females. Mitochondrial PBMC function was significantly higher in PBMCs from high-fit than from low-fit females, which was unrelated to PBMC composition and not impacted by a recent bout of exercise. Our study reveals a link between PBMC metabolism and levels of aerobic fitness, increasing the relevance of PBMC metabolism as a marker to study the impact of lifestyle factors on human health.

**NEW & NOTEWORTHY** Mitochondrial metabolism was significantly higher in PBMCs from high-fit than from low-fit females. This was unrelated to PBMC composition and not impacted by a recent bout of exercise. Our study reveals a link between PBMC metabolism and levels of aerobic fitness, increasing the relevance of PBMC metabolism as a marker to study the impact of lifestyle factors on human health.

## Graphical abstract

**Figure:**
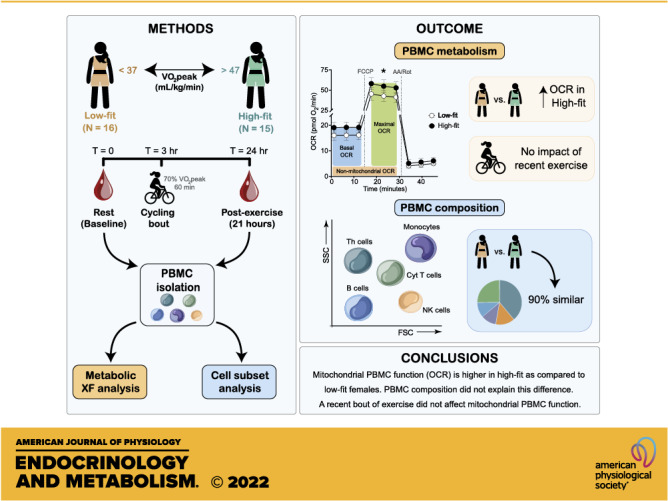

## INTRODUCTION

Lifestyle factors play a dominant role in health maintenance and the prevention of chronic diseases, such as type 2 diabetes (1), cardiovascular disease (2), and cancer (3). Adopting a healthy lifestyle that includes regular physical activity, a balanced diet, a healthy body mass index (BMI), and avoidance of smoking and alcohol intake, is associated with a lower chronic disease risk (4). Many biomarkers are available to assess the impact of lifestyle factors on chronic disease risk or disease progression (5), yet biomarkers that assess how lifestyle factors can contribute to optimizing human health have been less investigated. To this end, accurate, reproducible, clinically relevant, and sensitive biomarkers are needed, especially because metabolic differences between healthy individuals are smaller as compared with differences observed during disease pathology.

Immune cells are highly dynamic and reactive to acute environmental signals, such as infectious stimuli, but they can also respond to chronic lifestyle factors, such as physical activity (6), diet (7), and smoking (8). Peripheral blood mononuclear cells (PBMCs) are a readily accessible source of live immune cells from individuals and have been found to provide predictive disease markers for metabolic disorders, including obesity (9, 10). In addition, PBMCs have been used as surrogate tissue to monitor nutritional and metabolic responses (10, 11). Using animal models, it was shown that PBMCs can display specific metabolic alterations in response to, e.g., unbalanced diets (12) and fasting and refeeding (13). Since PBMCs could thus memorize or reflect metabolic alterations and can be readily obtained from individuals, they are of particular interest for studying the effect of lifestyle and lifestyle interventions on health outcomes in humans.

PBMCs are white blood cells with a single round nucleus that comprise several immune cell classes, including T- and B-lymphocytes, natural killer (NK) cells, monocytes, and dendritic cells (14, 15). Typically, 70%–90% of the PBMCs are lymphocytes, 10%–20% are monocytes, and 1%–2% are dendritic cells. Cell type frequencies within the lymphocyte population include 70%–85% cluster of differentiation (CD)3^+^ T cells, 5%–10% B cells, and 5%–20% NK cells. The CD3^+^ T cells are composed of CD4^+^ and CD8^+^ T cells, roughly in a 2:1 ratio. Different PBMC subsets do not only provoke different immune responses but they can also display distinct metabolic states (16–18). Importantly, apart from providing energy substrates and metabolic building blocks for immune cell proliferation and synthesis of macromolecules (e.g., cytokines), metabolic pathways have recently been shown to not only act as a consequence but also as driver of immune cell differentiation (16), which further increases the relevance of monitoring cellular metabolism as a biomarker for health. PBMCs are sampled with relative ease, low invasiveness, and high viability (19) and are thus highly suitable for metabolic profiling of immune cells ex vivo. A further advantage of PBMCs is their immediate metabolic response that is seen upon activation (20), which enables the assessment of metabolic flexibility on top of steady-state metabolic analysis and determination of metabolic capacity.

PBMC metabolism was found to be altered in several disease conditions, including diabetes type 2 (21), cardiovascular diseases (22), and obesity (23), and could possibly also be used as a cellular metabolic read-out of lifestyle factors and targeted lifestyle interventions for optimizing human health. To evaluate this potential in healthy individuals, we should better understand how longer-term lifestyle factors impact PBMC metabolism, and whether this is affected by a short-term lifestyle intervention. For example, previous studies have shown that PBMC metabolism not only responds to longer-term exercise training (24) but also to an acute exercise challenge (25). Since a single exercise bout often elicits a transient proinflammatory response whereas regular bouts of exercise induce an anti-inflammatory state (6, 26), the impact of regular physical activity (long-term effect) and a single-exercise session (short-term effect) on PBMC metabolism could differ, yet this has not been studied in detail. Furthermore, it is not yet clear whether the metabolic responses in PBMCs upon these exercise interventions are primarily related to alterations in cellular energy metabolism per se, or that these responses reflect changes in PBMC composition, since changes in PBMC subset frequencies have been demonstrated in response to exercise (27–29), and PBMC subsets are not only immunologically but also metabolically distinct (17, 18). Better understanding of the variation in PBMC subsets and overall PBMC composition between healthy donors and their contribution to the overall metabolic outcomes in PBMCs is therefore also needed.

We investigated in females how PBMC metabolism and PBMC composition are affected by high- and low levels of aerobic fitness, i.e., a difference in longer-term physical activity, by comparing endurance-trained (high-fit) and untrained (low-fit) females at baseline and after a recent bout of exercise (21 h before blood sampling). This study will improve our understanding of the use of PBMC metabolism as a biomarker to study the impact of lifestyle factors and lifestyle interventions on human health.

## MATERIALS AND METHODS

### Ethical Approval and Study Registration

The protocol for collection and handling of human samples was ethically approved by the medical ethical committee of Wageningen University and Research with Reference No. NL70136.081.19 and registered in the Dutch trial register (NL7891) on 23 July 2019. All procedures performed were in accordance with the ethical standards of the institutional and/or national research committee and with the 1964 Helsinki declaration. Written informed consent was obtained from all individual subjects included in the study.

### Study Subjects

Healthy young females (18–28 yr of age, BMI 18.5–25 kg/m^2^) were recruited from the local university and community population. Exclusion criteria were as follows: history of cardiovascular, respiratory, hematological, or metabolic disease; use of prescribed chronic medication; anemia (hemoglobin concentration < 12 g/dL); blood donation within 2 mo before the start of the study; smoking (>5 cigarettes/wk); recreational drug use or over the counter drug use during the study; use of performance-enhancing supplements; pregnancy or lactating. The use of oral contraceptives was not excluded; only the use of monophasic oral contraceptives containing low synthetic estradiol and progesterone was allowed and was controlled for (*n* = 7 in the high-fit and *n* = 6 in the low-fit group). Subjects were selected if they had a V̇o_2peak_ ≥ 47 mL/kg/min (high-fit group) or ≤37 mL/kg/min (low-fit group) determined using a maximal exercise test, measured using the validated screening protocol of Lagerwaard et al. (30, 31), which minimized the risk for selective bias. The V̇o_2peak_ cut-offs were based on previous findings from our laboratory in high-fit (trained) and low-fit (untrained) males (31) and previous studies in high-fit (trained) and low-fit (untrained) females (32–34). Sixteen high-fit and sixteen low-fit subjects were included. A total of 111 exercise tests were performed to end up with the desired sample size. One subject was excluded due to medication intake. For sufficient power, we focused on one sex, because PBMC metabolism was found to differ between males and females (35) and previous exercise studies have been mainly performed in males (36–38). The V̇o_2peak_ data and results of skeletal muscle mitochondrial capacity of these included subjects have been published previously by our group (30).

### Study Design

Subjects refrained from heavy physical exercise 48 h before the first study day and from any physical exercise and alcohol consumption 24 h before the first study day. Subjects adhered to dietary guidelines 24 h before each study day, which included the consumption of a standardized evening meal (73% carbohydrates/16% protein/11% fat, 1,818 kJ) before 8:00 PM and dietary guidelines for the consumption of breakfast, lunch, drinks, and snacks. After an overnight fast, blood was collected in the morning of the first study day (= baseline timepoint) and on the morning of the second study day, i.e., 21 h after a single bout of exercise (= postexercise timepoint). Blood samples (5 × 10 mL) were collected by venipuncture in vacutainers containing dipotassium (K2-) ethylenediaminetetraacetic acid (EDTA) (K2-EDTA, BD Biosciences, Vianen, The Netherlands, 367525) as anticoagulant and processed within 30 min after blood collection. Body fat percentage was measured according to the four-site method by Durnin–Womersley using the skinfold measurements of the triceps, biceps, sub scapula, and supra iliac, measured using a skinfold caliper (Harpenden, UK). Subjects received breakfast and after 2 h, subjects completed an individualized exercise protocol consisting of 60-min cycling on an electrically braked bicycle ergometer (Corival CPET, Lode, The Netherlands) at a workload aiming to equal 70% of their V̇o_2peak_. Oxygen consumption, carbon dioxide production, and air flow were measured using MAX-II metabolic cart (AEI technologies, Landivisiau, France). Exhaled air was continuously sampled from a mixing chamber and averaged over 15-s time windows. Oxygen consumption was measured in the first and last 15 min of the exercise test and used to confirm the relative oxygen consumption. If needed, small adjustments in workload were made to reach 70%. After the exercise protocol, subjects went home and refrained from moderate to heavy physical activity, kept low levels of light physical activity, and refrained alcohol consumption until blood collection on the second study day. The habitual dietary intake of the study subjects was determined via a validated food frequency questionnaire (FFQ) that assessed dietary intake in the past 4 wk (39). The self-reported diets of the high-fit and low-fit subjects were similar with no significant differences in total daily energy intake, carbohydrate intake, protein intake, or fat intake (Supplemental Fig. S1; see https://doi.org/10.6084/m9.figshare.17111537).

### Chemicals

Carbonyl cyanide-*p*-trifluoromethoxyphenylhydrazone (FCCP, C2920), oligomycin (OM, O4867, antimycin A (AA, A8674), rotenone (Rot, R8875), monensin sodium salt (MON, M5273), 2-deoxyglucose (2-DG, D6134), Concanavalin A (Con A, C2010), bovine serum albumin (BSA, A6003), sodium chloride (S9888) and Roswell Park Memorial Institute (RPMI) 1640 medium without phenol red and HEPES (11835030), Dulbecco’s phosphate-buffered saline (DPBS, 14190094), and Hanks’ Balanced Salt Solution (HBSS, 14175095) were purchased from Thermo Fisher Scientific (Pittsburgh, PA). Extracellular flux (XF) RPMI assay medium pH 7.4 (103576-100), XF 1.0 M glucose (103576-100), XF 100 mM pyruvate (103578-100), and XF 200 mM glutamine (103579-100) were purchased from Seahorse Biosciences, Agilent Technologies (Santa Clara, CA).

### PBMC Isolation

EDTA-collected blood (50 mL ± 1 mL, except for two samplings where we took 48 and 45 mL blood) was diluted with DPBS (1×) without magnesium and calcium supplemented with sodium citrate buffer (1% vol/vol) as anticoagulant in a 1:1 ratio. Diluted blood was carefully poured into Leucosep tubes (Thermo Fisher Scientific, 227289) that were filled with Ficoll Paque Plus (15 mL, GE Healthcare, Marlborough, MA, 17144003), followed by density gradient centrifugation for 10 min at 1,000 *g* at RT with acceleration five and zero braking. The PBMC fraction was collected using sterile Pasteur pipettes and centrifuged for 10 min at 600 *g* at RT with brake to concentrate the PBMC layer and facilitate removal of residual Ficoll and plasma. Supernatant was discarded, and cells were washed three times using DPBS (1×, 20 mL) without magnesium and calcium, supplemented with sodium citrate buffer (1% vol/vol) and fetal bovine serum (FBS, 2% vol/vol, DPBS-1% citrate-2% FBS) and centrifuged for 7 min at 250 *g* at RT. Supernatant was discarded, and cells were resuspended in RPMI 1640 medium without phenol red and HEPES (30 mL, Thermo Fisher Scientific, 11835030), supplemented with FBS (10% vol/vol). Total PBMC number and PBMC viability was determined for each donor in a 1:10 dilution using acridine orange and propidium iodide staining (ViaStain, Nexcelom Bioscience, Lawrence, MA, CS2-0106) in a Nexcelcom Cell Counter (Nexcelcom Bioscience) in fluorescent mode (*n* = 8). PBMCs were directly used for XF assay measurements or cryopreserved and stored in liquid nitrogen for later fluorescence-activated cell sorting (FACS) staining and analysis. For cryopreservation, 6 × 10^6^ PBMCs were washed with DPBS-1% citrate-2% FBS and centrifuged for 5 min at 400 *g* at RT. Supernatant was discarded and cells were resuspended with ice-cold FBS (1 mL), followed by gentle droplet wise addition of ice-cold FBS-DMSO (80%/20% vol/vol, 1 mL) to achieve a final FBS-DMSO concentration of 90%/10% vol/vol and transferred to cold cryovials (3 × 10^6^ PBMCs per vial). Cryovials were inserted in a cold (4°C) isopropanol chamber, stored overnight at −80°C, and transferred to liquid nitrogen the next day.

### High-Contrast Bright-Field Imaging and Image Analysis

High-contrast bright-field images were obtained before the XF assay run using the Cytation 1 Cell Imaging Multi-Mode Reader (BioTek, Winooski, VT) followed by image analysis according to our previously published “R-integrated pixel intensity (PIXI) analysis” protocol for normalization of XF assay data (41).

### XF Analysis with Seahorse XFe96 Analyzer

Oxygen consumption rate (OCR) and proton efflux rate (PER) measurements were performed in a Seahorse extracellular flux (XF)e96 Analyzer (Seahorse Biosciences). PBMCs (225,000) per well (*n* = 8) were plated onto Cell-Tak (Corning, NY, 354240) coated XF96 cell plates in XF RPMI assay medium pH 7.4 (50 μL, XF assay medium) supplemented with XF glucose (11 mM), XF pyruvate (1 mM), and XF glutamine (2 mM) and left for 10 min at RT. In addition, 75–300,000 PBMCs per well (*n* = 3 or 4) were plated for the internal calibration curve that was used for normalization. Cell plate was centrifuged for 1.5 min at 200 *g* at RT with acceleration one and zero braking. Afterward, an additional volume of XF assay medium (130 μL) was added to the cells and cells were incubated for 30 min at 37°C without CO_2_. XF measurements included two injection strategies (*A* and *B*) that were used in parallel. *Injection strategy A* included serial injections of FCCP (1.25 μM), AA plus Rot (AA/Rot, 2.5 μM/1.25 μM), and 2-DG (50 mM) and *injection strategy B* included serial injections of OM (1.5 μM), AA/Rot (2.5 μM/1.25 μM), and MON (20 μM). These injection strategies were preceded by injection with XF assay medium (for nonactivated, control PBMCs) or Con A (25 μg/mL, for real-time activated PBMCs). The total XF assay protocol consisted of 18 measurement cycles of which each cycle included 5 min of which 2 min mixing, 0 min waiting, 3 min measuring. The first injection (XF assay medium or Con A) was inserted after three measurement cycles and was followed by six measurement cycles without injection to allow PBMC activation. Each injection strategy (*A* with XF assay medium, *A* with Con A, *B* with XF assay medium, *B* with Con A) included 2–8 technical replicates per subject. The time window (from isolated PBMCs until the start of the Seahorse assay) was on average 180 ± 26 min. Glycolytic PER (glycoPER) values were calculated by subtracting mitochondrial PER (= OCR × 0.61) values from total PER values to correct for the contribution of mitochondrial-derived CO_2_ production to acidification of the XF assay medium (42) and reported as glycolytic rate (GR). OCR and GR values were normalized against the number of PIXI analyzed cells (41).

### Flow Cytometry Staining and Analysis

PBMCs were stained with fluorochrome-conjugated antibodies against extracellular markers for cell phenotyping (Table 1). Every antibody was titrated individually within the recommended range (4, 2, 1, 0.5, and 0 µL/sample). The staining index (SI) was calculated, and the highest value was selected as the optimal concentration for FACS staining. The optimal antibody concentrations are presented in Supplemental Table S1; see https://doi.org/10.6084/m9.figshare.17125031. For FACS staining, 3.0 × 10^6^ PBMCs were quickly thawed in a 37°C water bath under continuous agitation and transferred to a pre-filled 50-mL tube containing RPMI-1640 medium supplemented with FBS (20% vol/vol, 10 mL). Cell suspension was centrifuged for 10 min at 300 *g* at RT and the supernatant was discarded. Cells were washed twice with RPMI-1640 with FBS (20% vol/vol, 10 mL) and centrifuged for 10 min at 300 *g* at RT. Supernatant was discarded, and cells were resuspended in RPMI-1640 with FBS (20% vol/vol, 500 µL). PBMCs (1.5 × 10^6^) per well (250 µL) was added to a 96-well NUNC plate (Thermo Fisher Scientific, 267245) and cells were washed by adding DPBS (1×, 100 µL) followed by plate centrifugation for 4 min at 400 *g* at RT. Supernatant was discarded and cells were washed again with DPBS (1×, 200 µL) and the plate was centrifuged for 4 min at 400 *g* at RT. For live/dead staining, Zombie near-infrared (NIR) viability solution (Biolegend, San Diego, CA, 423105) was 500 times diluted in DPBS and added (100 µL) followed by incubation for 25 min at RT, in the dark. Supernatant was discarded and cells were washed with FACS buffer (200 µL) containing DPBS (1×) supplemented with BSA (0.5% vol/vol), EDTA (2.5 mM), and sodium azide (NaN_3_, 10% vol/vol) (final pH 7.4). The plate was centrifuged for 3 min at 400 *g* at RT and fluid was discarded. The mixture of antibodies (Table 1) diluted in FACS buffer was added to the cells and the plate was incubated for 30 min at 4°C in the dark. Cells were washed three times by adding cold FACS buffer (200 µL) and plate centrifugation for 3 min at 400 *g* at 4°C. Supernatant was discarded, and the cell pellet was resuspended in FACS buffer (300 µL) and measured on CytoFLEX LX (Beckman Coulter’s, Indianapolis, IN). The generated flow cytometry data were analyzed using Flowjo v10 (FlowJo LLC, Ashland, OR, RRID:SCR_008520). Cell frequencies are depicted as the percentage CD45^+^NIR^−^ leukocytes (% viable leukocytes) determined by live/dead staining.

**Table 1.** Fluorochrome-conjugated antibodies used for FACS staining

Antibody	Fluorochrome	Reactivity	Light Chain	Clone	Company*	Catalog Number	RRID
α-CD3	AF700	Human	κ	UCHT1	Biolegend	300424	AB_493741
α-CD4	BV510	Human	κ	OKT4	Biolegend	317444	AB_2561866
α-CD8a	BV650	Human	κ	RPA-T8	Biolegend	301042	AB_2563505
α-CD25	BV421	Human	κ	BC96	Biolegend	302630	AB_11126749
α-CD127	APC	Human	κ	A019D5	Biolegend	351342	AB_2564137
α-CD14	BV605	Human	κ	63D3	Biolegend	367126	AB_2716231
α-HLA-DR	FITC	Human	κ	L243	Biolegend	307632	AB_1089142
α-CD56	PE	Human	κ	5.1H11	Biolegend	362524	AB_2564161
α-CD19	PE/Cy7	Human	κ	HIB19	Biolegend	302216	AB_314246
α-CD20	PE/Daz594	Human	κ	2H7	Biolegend	302348	AB_2564387
α-CD45	PerCp	Human	κ	2D1	Biolegend	368506	AB_2566358

CD, cluster of differentiation; HLA-DR, human leukocyte antigen-DR isotype.

* All antibodies were developed, produced, and distributed by Biolegend.

### Statistical Analyses

Statistical analyses were performed using IBM SPSS Statistics for Windows (Version 25.0, IBM Corp, Armonk, NY, RRID:SCR_002865). Graphs were created using GraphPad Prism (Version 8.0, Graphpad Software, CA, RRID:SCR_002798). Normality was checked using Shapiro–Wilk normality tests. Data are presented as means ± standard deviation (SD) for normally distributed data and as median [interquartile range (IQR)] for not normally distributed data. Not normally distributed data was log-transformed for statistical analysis. Repeated-measures ANOVA (RM-ANOVA) was used to study the effect of fitness level (between-subjects factor) and the effect of a recent bout of exercise or ex vivo Con A stimulation (within-subjects factors) on the response of XF assay parameters and the interaction between them. All assumptions for RM-ANOVA were met. Two-sided unpaired Student’s *t* tests were used to compare the normally distributed subject characteristics and the relative Con A-induced differences between high-fit and low-fit females. Mann–Whitney *U* tests were used to compare not normally distributed subject characteristics. *P* values <0.05 were considered as statistically significant.

## RESULTS

### Total PBMC Number is Not Affected by Fitness Level, But Lower upon a Recent Bout of Exercise

PBMCs were isolated from females with high (V̇o_2peak_ ≥ 47 mL/kg/min, *n* = 15) and low (V̇o_2peak_ ≤ 37 mL/kg/min, *n* = 16) levels of aerobic fitness representing trained, physically active females and untrained, low physically active females, respectively. Subject characteristics are shown in Table 2. The total number of isolated PBMCs per total blood volume was not significantly different between high-fit (102.5 ± 29.6 × 10^6^ cells) and low-fit (104.3 ± 27.1 × 10^6^ cells) females (*P*_group_ = 0.829) and significantly lower after the recent bout of exercise in both groups (95.2 ± 22.2 × 10^6^ cells and 97.04 ± 18.1 × 10^6^ cells respectively, *P*_exercise_ = 0.036), but again not different between the two groups (*P* = 0.981). All cell viabilities were >97% and not significantly different between high-fit (97.6 ± 0.5%) and low-fit (97.8 ± 0.4%) females (*P*_group_ = 0.182) and not significantly impacted by the recent bout of exercise (*P*_exercise_ = 0.580).

**Table 2.** Subject characteristics

	Low-Fit (*n* = 16)	High-Fit (*n* = 15)
Age, yr	24.0 [21.3–25.5]	21.8 [21.6–23.7]
Ethnicity	Caucasian (11), Asian (1), Indo-pacific (4)	All Caucasian
Weight, kg	59.2 ± 7.2	61.2 ± 7.0
Height, m	1.63 ± 0.08*	1.68 ± 0.05*
Fat mass (% of weight)	28.9 ± 3.9*	25.1 ± 4.4*
V̇o_2peak_, mL/kg/min	35.1 [32.2–35.7]****	50.4 [49.0–54.0]****
Baecke total score	7.3 ± 1.0****	9.5 ± 0.8****
Hemoglobin, mmol/L	8.4 ± 0.6	8.5 ± 0.6

V̇o_2peak_, maximal oxygen consumption values. Values are means ± SD for normally distributed data, and median [IQR] for not normally distributed data.

*P < 0.05; ****P < 0.0001.

### The Overall PBMC Composition is Not Significantly Affected by Fitness Level and a Recent Bout of Exercise

**Figure 1.**
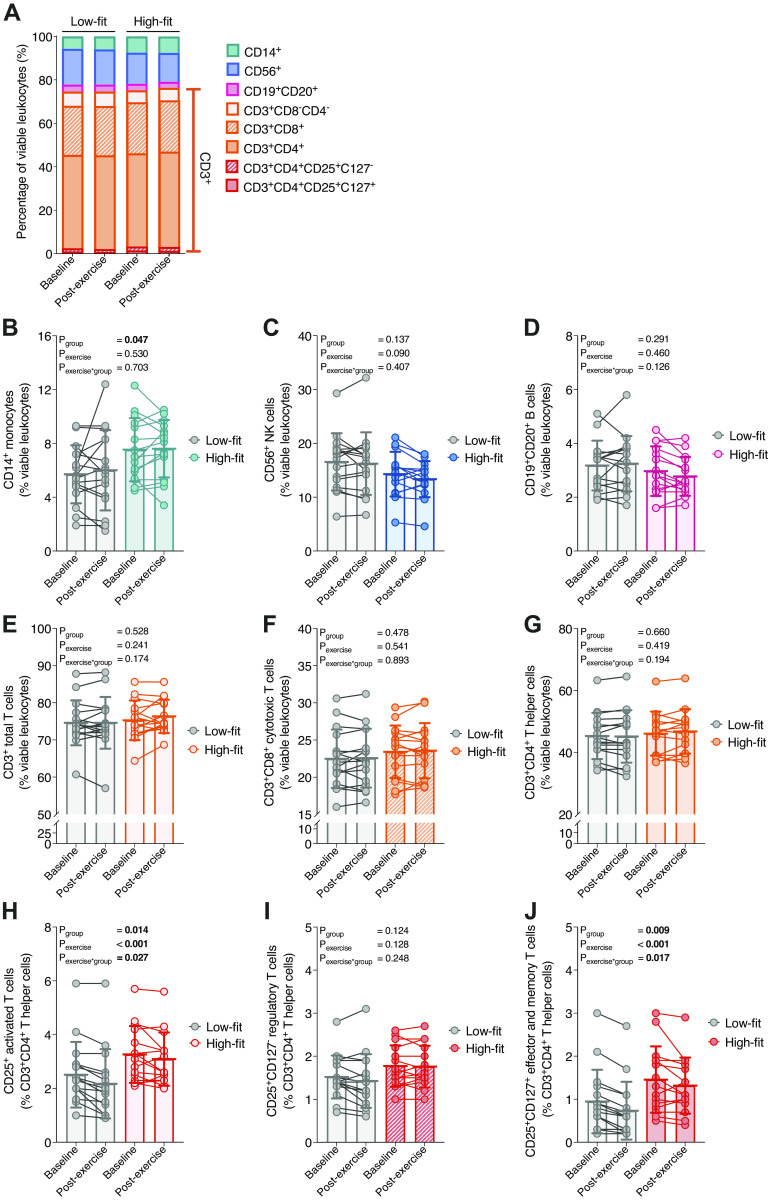
Characterization of peripheral blood mononuclear cell (PBMC) types in high-fit and low-fit females at baseline and after a recent bout of exercise. Stacked (*A*) and single percentages of CD14^+^ (*B*), CD56^+^ (*C*), CD19^+^CD20^+^ (*D*), CD3^+^CD8^−^CD4^−^ (*E*), CD3^+^CD8^+^ (*F*), CD3^+^CD4^+^ (*G*), CD3^+^CD4^+^CD25^+^ (*H*), CD3^+^CD4^+^CD25^+^CD127^−^ (*I*), and CD3^+^CD4^+^CD25^+^CD127^+^ (*J*) subsets relative to the total number of viable PBMCs (%) at baseline and postexercise in low-fit (*n* = 16, *left* in stacked plot, gray in single plots) and high-fit (*n* = 15, *right* in stacked plot, colored in single plots) females. Main effects (fitness level and recent exercise) and interaction effects were analyzed using repeated-measures ANOVA (RM-ANOVA). Significant *P* values (<0.05) are indicated in bold.

To investigate how physical fitness level is related to metabolic PBMC activity, we first characterized various cell subsets in PBMCs from high- and low-fit females and studied whether these subsets are affected by a recent bout of exercise. We used lineage-specific antibodies against T cells, B cells, NK cells, and monocytes (Supplemental Fig. S2; see https://doi.org/10.6084/m9.figshare.16817437) and found that the overall PBMC composition was similar between high-fit and low-fit females at baseline as well as after a recent bout of exercise (Fig. 1*A*). The number of viable leukocytes (CD45^+^NIR^−^ leukocytes) was >90% after thawing. In line with overall similar PBMC composition, subset analysis at baseline showed that frequencies of CD56^+^NK cells, CD19^+^CD20^+^ B cells, and CD3^+^ total T cells, including CD3^+^CD4^+^ T helper (Th) cells and CD3^+^CD8^+^ cytotoxic T cells, were not significantly different between PBMCs from high-fit and low-fit females (*P*_group_ = 0.137, 0.291, 0.528, 0.478, and 0.660, respectively). We did observe that PBMCs from high-fit and low-fit females significantly differed in frequencies of CD14^+^ monocytes (7.52 ± 2.35% in high-fit compared with 5.69 ± 2.15% in low-fit females, *P*_group_ = 0.047) and of CD3^+^CD4^+^CD25^+^-activated T cells [3.10% (2.30%–4.20%) in PBMCs from high-fit compared with 2.35% (1.70%–2.90%) in PBMCs from low-fit females, *P*_group_ = 0.014, Fig. 1, *B*–*H*). When co-expression of CD127 and CD25 was analyzed, PBMCs from high-fit and low-fit females did not significantly differ in the frequencies of CD3^+^CD4^+^CD25^+^CD127^−^ Treg cells (1.77 ± 0.48% in high-fit compared with 1.52 ± 0.50% in low-fit females, *P*_group_ = 0.124, Fig. 1*I*), yet frequencies of CD3^+^CD4^+^CD25^+^CD127^+^ non-Treg cells, i.e., effector and memory T cells, were significantly different between PBMCs from high-fit (1.30 [0.80%–1.90%]) compared to PBMCs from low-fit (0.75% [0.38%–1.25%]) females, *P*_group_ = 0.009 Fig. 1*J*]. Although, there is a 24% difference in CD14^+^ monocyte frequency between the two groups, just like the CD3^+^CD4^+^CD25^+^-activated T cells, they constitute a relative minor part of the total PBMC fraction (<10%), and thus changes are likely not substantially affecting the overall PBMC composition. PBMC cell type frequencies were also not significantly altered after a recent exercise bout (*P*_exercise_ > 0.05 for all, Fig. 1, *B*–*G* and *I*), except for the small subset of CD3^+^CD4^+^CD25^+^-activated T cells (*P*_exercise_ < 0.001, Fig. 1*H*) and CD3^+^CD4^+^CD25^+^CD127^+^ T cells (*P*_exercise_ < 0.001, Fig. 1*J*).

### Baseline and Postexercise Mitochondrial Function is Significantly Higher in PBMCs from High-Fit Compared with Low-Fit Females

**Figure 2.**
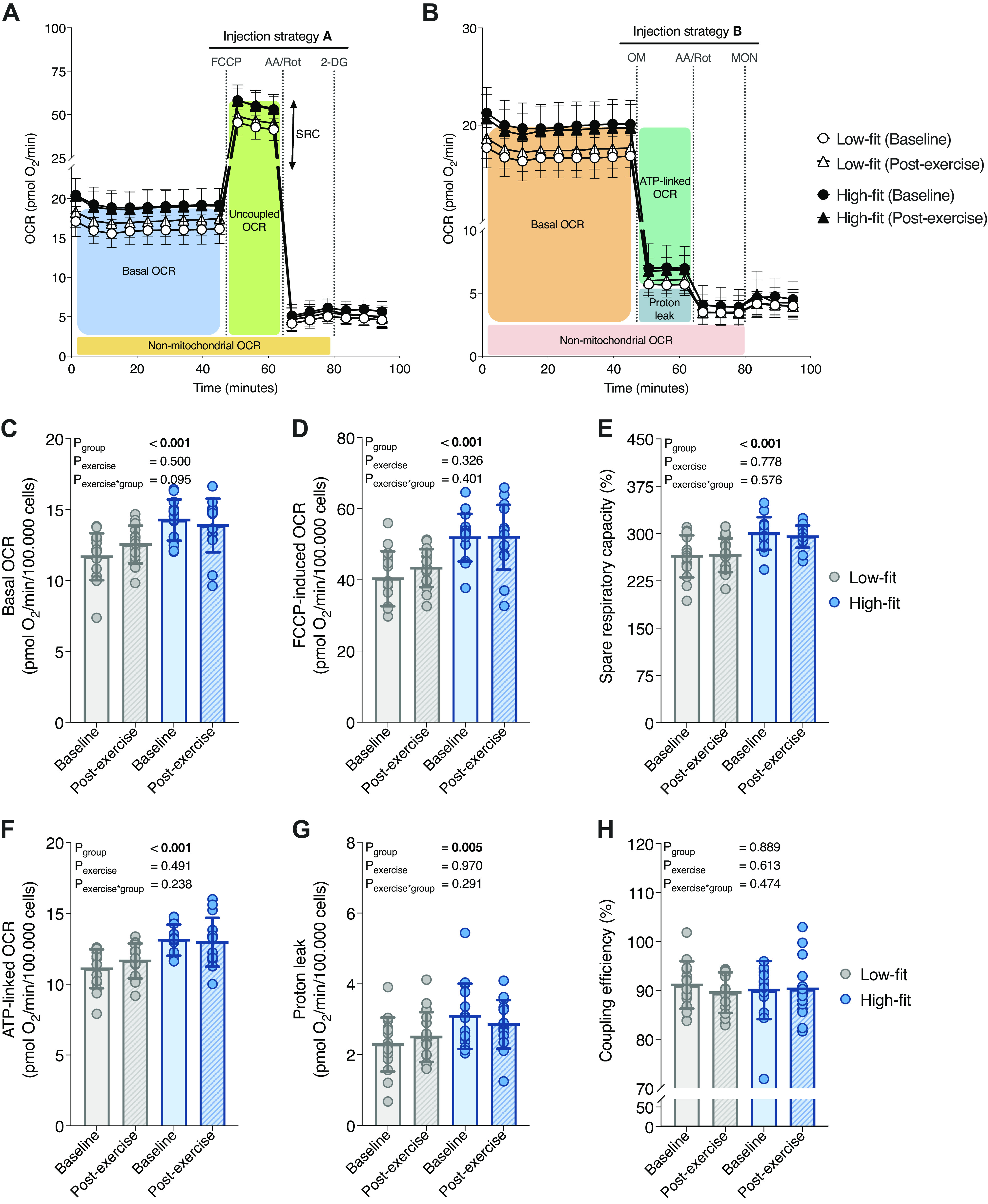
Mitochondrial peripheral blood mononuclear cell (PBMC) function in high-fit and low-fit females at baseline and after a recent bout of exercise. Representation of mitochondrial parameters derived from the extracellular flux (XF) assay using *injection strategy A* (*A*) or *B* (*B*) and traces for low-fit (*n* = 16, white) and high-fit (*n* = 15, black) at baseline (dots) and postexercise (triangles). Basal oxygen consumption rate (OCR) (*C*), FCCP-induced OCR (*D*), and corresponding spare respiratory capacity (*E*). ATP-linked OCR (*F*), proton leak (*G*), and corresponding coupling efficiency (*H*). Parameters are calculated for low-fit (*n* = 16, gray) and high-fit (*n* = 15, blue) females at baseline (clear bars) or postexercise (striped bars). Values are depicted per 10^5^ R-integrated pixel intensity (PIXI) analyzed cells for absolute rates or as a percentage (%) for ratios of corresponding values. Main effects (fitness level and recent exercise) and interaction effects were analyzed using repeated-measures ANOVA (RM-ANOVA). Significant *P* values (<0.05) are indicated in bold.

**Figure 3.**
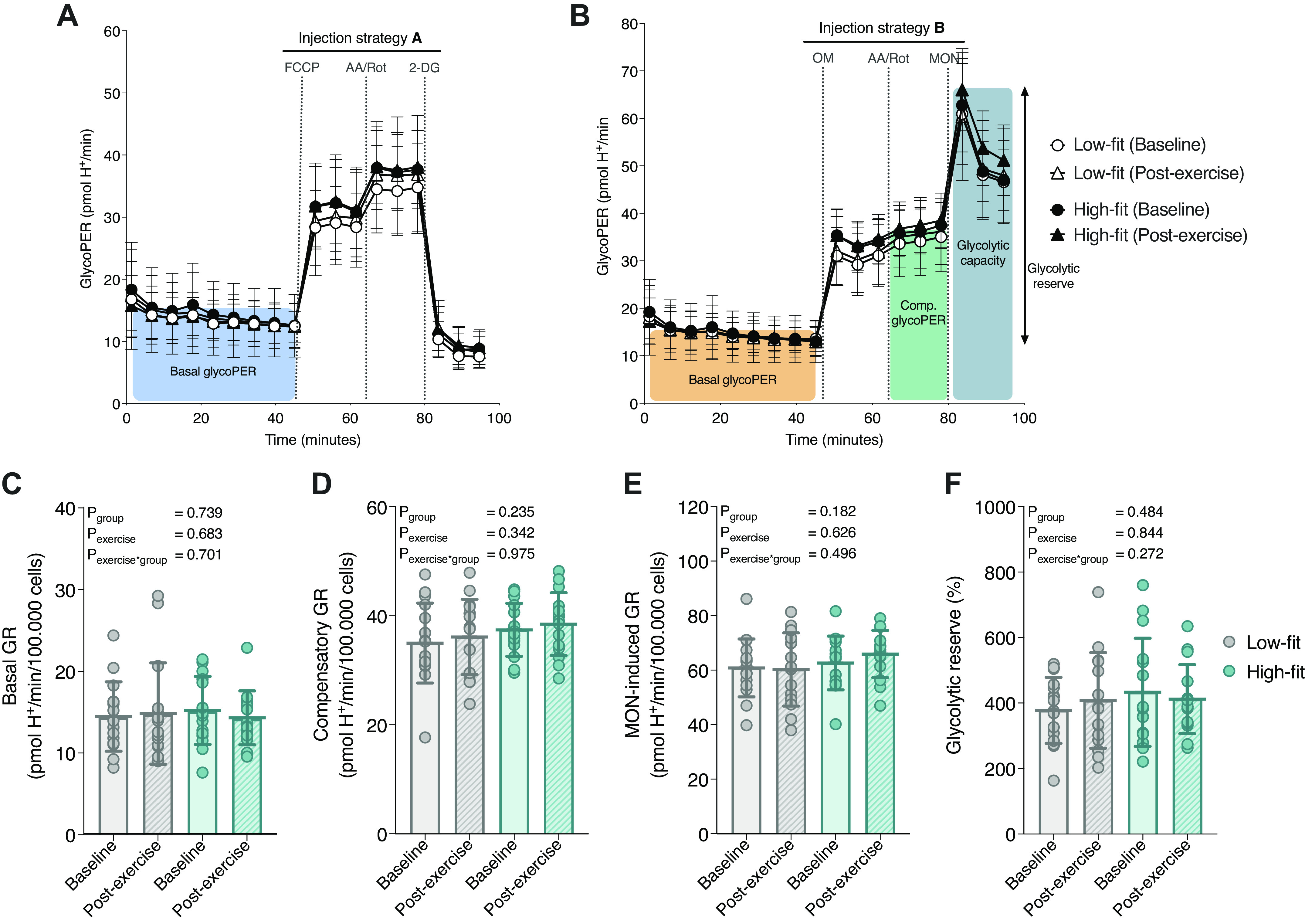
Glycolytic peripheral blood mononuclear cell (PBMC) function in high-fit and low-fit females at baseline and after a recent bout of exercise. Representation of glycolytic parameters derived from the extracellular flux (XF) assay using *injection strategy A* (*A*) or *B* (*B*) and traces for low-fit (*n* = 16, white) and high-fit (*n* = 15, black) females at baseline (dots) and postexercise (triangles). Basal glycolytic rate (GR, *C*), compensatory GR (*D*), monensin (MON)-induced GR (*E*), and the glycolytic reserve calculated as the ratio between MON-induced GR and basal GR (*F*). Parameters are calculated for low-fit (*n* = 16, gray) and high-fit (*n* = 15, turquoise) females at baseline (clear bars) or postexercise (striped bars). Values are depicted per 10^5^ R-integrated pixel intensity (PIXI) analyzed cells for absolute rates or as a percentage (%) for ratios of corresponding values. Main effects (fitness level and recent exercise) and interaction effects were analyzed using repeated-measures ANOVA (RM-ANOVA). Significant *P* values (<0.05) are indicated in bold.

Next, we aimed to study whether PBMCs from high-fit and low-fit females are metabolically different by analyzing oxidative and glycolytic metabolism using XF analysis. We used a standardized experimental setup that was validated for low technical variation and sampling of many individuals on multiple days (41). Using two different injection strategies, *injection strategy A* (Figs. 2*A* and 3*A*) and *injection strategy B* (Figs. 2*B* and 3*B*), we were able to probe a complete set of parameters of mitochondrial and glycolytic function and capacity, in a relatively short-run time. PBMCs from high-fit females had significantly higher basal OCR (14.23 ± 1.44 pmol O_2_/min/10^5^ cells) compared with PBMCs from low-fit females (11.65 ± 1.65 pmol O_2_/min/10^5^ cells, *P*_group_ < 0.001, Fig. 2*C*). Similarly, FCCP-induced OCR was significantly higher in PBMCs from high-fit (52.01 ± 6.79 pmol O_2_/min/10^5^ cells) compared with PBMCs from low-fit (40.36 ± 7.64 pmol O_2_/min/10^5^ cells) females (*P*_group_ < 0.001, Fig. 2*D*). Since spare respiratory capacity (SRC) was also significantly higher in PBMCs from high-fit (300 ± 26%) compared with PBMCs from low-fit (264 ± 33%) females (*P*_group_ < 0.001, Fig. 2*E*), mitochondrial respiratory capacity was significantly higher in PBMCs from high-fit females. Adenosine triphosphate (ATP)-linked OCR and mitochondrial proton leak were significantly higher in PBMCs from high-fit females (13.10 ± 1.10 pmol O_2_/min/10^5^ cells and 3.09 ± 0.92 pmol O_2_/min/10^5^ cells, respectively) compared with PBMCs from low-fit females (11.09 ± 1.37 pmol O_2_/min/10^5^ cells and 2.29 ± 0.76 pmol O_2_/min/10^5^ cells, *P*_group_ < 0.001 and *P* = 0.005, respectively) (Fig. 2, *F* and *G*). However, the percentage of mitochondrial respiration that is linked to ATP production, also defined as mitochondrial coupling efficiency, was not significantly different between high-fit [91.7% (88.7%–93.8%)] and low-fit [91.1% (87.2%–94.3%)] females (*P*_group_ = 0.889, Fig. 2*H*). The recent exercise bout allows us to analyze the effect of a single, short-term exercise intervention on PBMC metabolism, while simultaneously minimizing the impact of a temporary shift in PBMC subsets upon acute exercise, which has returned to baseline within 24 h after exercise completion (27–29). PBMC mitochondrial respiration and respiratory capacity were not significantly affected by the recent bout of exercise (*P*_exercise_ > 0.05 for all OCR parameters), and the exercise response did not significantly differ between high-fit and low-fit females (*P*_exercise × group_ > 0.05 for all OCR parameters (Fig. 2, *C–H*). Thus, mitochondrial function was significantly higher in PBMCs from high-fit as compared with low-fit females, at baseline as well as after a recent exercise intervention.

### Glycolytic Function Does Not Significantly Differ between PBMCs from High-Fit and Low-Fit Females

In a similar way, we studied whether parameters of glycolytic function were affected by fitness level and a recent bout of exercise. Basal GR was not significantly different between PBMCs from high-fit [14.58 (12.13–19.04) pmol H^+^/min/10^5^ cells] and low-fit [13.55 (11.19–16.31) pmol H^+^/min/10^5^ cells] females (*P*_group_ = 0.739, Fig. 3*C*). To assess PBMC glycolytic capacity, we first blocked mitochondrial respiration using AA and Rot injection, which forces the cells to upregulate their glycolytic machinery to compensate for the respiratory loss of ATP, which is therefore defined as compensatory glycolysis. Next, we injected the ionophore monensin (MON), which promotes cellular Na^+^ import and stimulates ATP hydrolysis by Na^+^/K^+^-ATPase (44), thereby further increasing cellular ATP demands and maximizing the glycolytic rate when mitochondrial respiration is fully blocked (45). We did not find a significant difference in compensatory GR, MON-induced GR, or glycolytic reserve between PBMCs from high-fit (37.45 ± 4.86 pmol H^+^/min/10^5^ cells, 62.76 ± 9.86 pmol H^+^/min/10^5^ cells, and 433 ± 165%, respectively) and PBMCs from low-fit females (35.02 ± 7.33 pmol H^+^/min/10^5^ cells, 60.94 ± 10.62 pmol H^+^/min/10^5^ cells, and 378 ± 101%, respectively, *P*_group_ = 0.235, 0.182, 0.484, Fig. 3, *D*–*F*). Furthermore, PBMC glycolytic function and capacity were not significantly affected by a recent bout of exercise (*P*_exercise_ > 0.05 for all GR parameters) and high-fit and low-fit females did not respond differently to exercise (*P*_exercise × group_ > 0.05 for all GR parameters (Fig. 3, *C–F*). Thus, glycolytic function parameters did not significantly differ between PBMCs from high-fit and low-fit females, and were not significantly affected by a recent bout of exercise.

### Acute Immunometabolic Stimulation of PBMCs with Con A Does Not Impact the Relative Differences in PBMC Metabolism between High-Fit and Low-Fit Females

**Figure 4.**
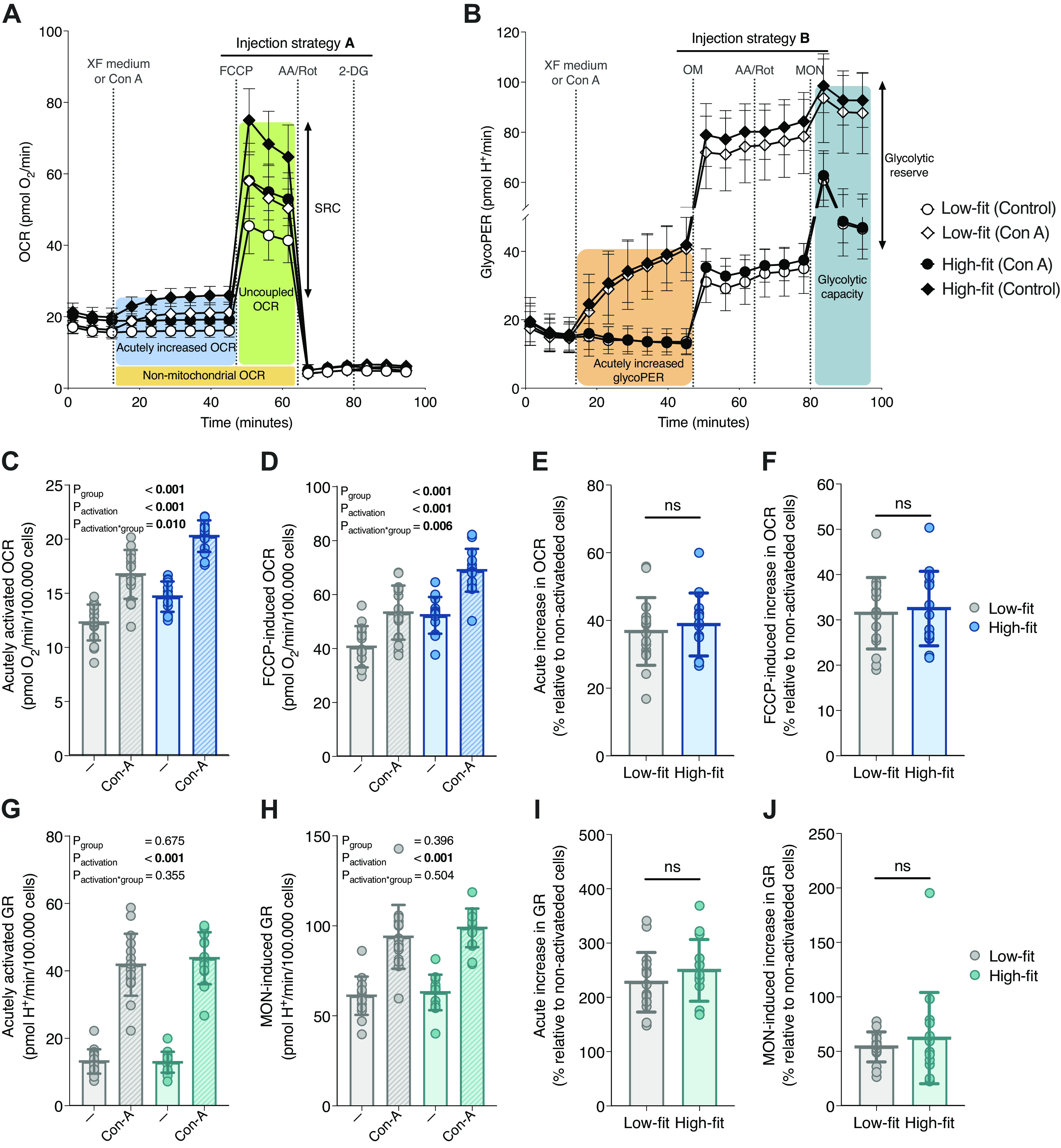
The effect of acute peripheral blood mononuclear cell (PBMC) stimulation on mitochondrial and glycolytic PBMC function in high-fit and low-fit females. Representation of the mitochondrial (*A*) and glycolytic (*B*) parameters derived from the induced extracellular flux (XF) assay using a first injection with XF assay medium for quiescent, control PBMCs (dots), or the mitogen Concanavalin A (Con A) for activated PBMCs (diamonds) followed by *injection strategy A* (*A*) or *B* (*B*) for low-fit (*n* = 16, white) and high-fit (*n* = 15, black) females. Acutely activated (*C*) and FCCP-induced (*D*) oxygen consumption rate (OCR) per 10^5^ R-integrated pixel intensity (PIXI) analyzed cells in control PBMCs (clear bars) or Con A-stimulated PBMCs (striped bars) from low-fit (*n* = 16, gray) and high-fit (*n* = 15, blue) females. The acute increase in OCR (*E*) and FCCP-induced OCR (*F*) in Con A-stimulated PBMCs compared with control PBMCs (%) in low-fit (*n* = 16, gray) and high-fit (*n* = 15, blue) females. Acutely activated (*G*) and monensin (MON)-induced (*H*) glycolytic rate (GR) per 10^5^ PIXI analyzed cells in control PBMCs (clear bars) or Con A-stimulated PBMCs (striped bars) from low-fit (*n* = 16, gray) and high-fit (*n* = 15, turquoise) females. The acute increase in GR (*I*) and MON-induced GR (*J*) in Con A-stimulated PBMCs compared with control PBMCs (%) in low-fit (*n* = 16, gray) and high-fit (*n* = 15, turquoise) females. Main effects (fitness level and Con A) and interaction effects were analyzed using repeated-measures ANOVA (RM-ANOVA) (C, *D*, *G*, and *H*) and the relative Con A-induced differences were analyzed using unpaired Student’s *t* tests (E, *F*, *I*, and *J*). Significant *P* values (<0.05) are indicated in bold. ns, not significant.

To study alterations in metabolic pathways in response to immune cell activation, PBMCs are often ex vivo stimulated with mitogenic compounds (20, 46–49). To investigate whether fitness level influences the acute metabolic switch that is seen upon mitogenic stimulation, we measured oxidative and glycolytic metabolism in in situ activated PBMCs and compared this to naïve, quiescent PBMCs. Concanavalin A (Con A) was used as a mitogenic lectin, which upregulates the respiratory and glycolytic machinery to provide the increased energy requirements that is necessary to support Con A-induced T cell activation and proliferation (20, 50), which includes for example enhanced ion signaling and increased cytokine synthesis (51, 52). Indeed, we demonstrated that acute PBMC stimulation with Con A upregulated mitochondrial respiration and glycolytic rate in PBMCs (Fig. 4, *A* and *B*). This resulted in a significant elevation of basal OCR (acutely activated OCR) and FCCP-induced OCR, in PBMCs from both high-fit and low-fit females (both *P*_activation_ < 0.001, Fig. 4, *C* and *D*). The absolute Con A induced increase in basal OCR and FCCP-induced OCR was significantly higher in high-fit compared with low-fit females (*P*_activation × group_ = 0.010 and 0.006, respectively, Fig. 4, *C* and *D*). When plotted relatively to nonactivated PBMCs, the Con A induced increase in basal OCR and FCCP-induced OCR did not significantly differ between the two groups (*P* = 0.558 and *P* = 0.725, respectively, Fig. 4, *E* and *F*). Similarly, Con A stimulation resulted in a significant increase in basal GR (acutely activated GR) and MON-induced GR, in PBMCs from both high-fit and low-fit females (both *P*_activation_ < 0.001, Fig. 4, *G* and *H*). Here, the absolute Con A induced increase in basal and MON-induced GR was not significantly different between high- and low-fit females (*P*_activation × group_ = 0.355 and 0.504, respectively, Fig. 4, *G* and *H*), neither were the relative increases (*P* = 0.285 and 0.473, respectively, Fig. 4, *I* and *J*). Thus, PBMCs from high-fit females showed higher mitochondrial respiration and capacity in response to Con A stimulation compared with low-fit females, but the relative metabolic response was not different between the two groups.

## DISCUSSION

This study demonstrates that high-fit females had significantly higher PBMC mitochondrial function parameters than low-fit females, whereas glycolytic parameters were not different. Furthermore, either a recent exercise challenge, or an ex vivo immunological challenge did not significantly alter metabolic function in isolated PBMCs. This indicated that especially long-term lifestyle differences can be imprinted in PBMC metabolism, whereas a short-term, recent exercise intervention is not reflected in PBMC metabolic functions. We also showed that the overall PBMC composition was similar between high-fit and low-fit females, i.e., the major contributing PBMC subsets were not significantly affected by fitness level or a recent bout of exercise.

We are the first study that revealed a link between levels of aerobic fitness and mitochondrial function in PBMCs, by showing that mitochondrial respiration and capacity was significantly higher in high-fit (trained) than low-fit (untrained or sedentary) young female adults, which was unrelated to immune cell composition. We also demonstrated that a recent bout of exercise did not alter PBMC mitochondrial function neither in high-fit nor in low-fit female individuals, indicating that a longer period of aerobic exercise training exposure might be required before the effects are reflected in metabolic PBMC profiles. This is in line with previous studies, which showed that 6 or 12 wk of aerobic exercise training increased mitochondrial function in respectively lymphocytes (36) and PBMCs (24) and found that protein and gene expression levels of mitochondrial markers in PBMCs were enhanced with 8 wk of aerobic exercise training (38), whereas a 2-wk aerobic exercise protocol did not alter mitochondrial function in PBMCs (37), indeed suggesting that longer-, but not shorter-term training status impacts PBMC mitochondrial function.

The bodily adaptations that occur upon regular exercise could possibly underlie our observation that PBMC metabolism is different between high-fit and low-fit females (53). Exercise triggers the release of biologically active proteins and metabolites by multiple tissues that are secreted into the systemic circulation and impact other organs and physiological systems (26, 54). For example, interleukin (IL)-6 is a cytokine that is acutely secreted from contracting skeletal muscle, which promotes the release of the anti-inflammatory cytokines IL-10 and IL-1 receptor antagonist (IL-1RA) and increases the mobilization of nutrients from liver and adipose tissue (55). Regular exercise has been suggested to lower the magnitude of the IL-6 response to exercise as well as resting IL-6 levels (55), and IL-6 levels were shown to negatively correlate with mitochondrial PBMC function (22, 23). Furthermore, serum lipid metabolite levels, such as triglycerides, fatty acids, and glycoproteins, were found to differ between high-fit and low-fit individuals (56). Since circulating lipid metabolites have been found to alter gene and protein expression levels of mitochondrial markers in isolated PBMCs (57) as well as ex vivo cytokine production in isolated PBMCs (58, 59), the altered metabolic make-up of the plasma upon regular aerobic exercise could imprint a long-lasting adaptation of PBMC mitochondrial function. Given that a single, recent exercise bout or an ex vivo immunological stimulus did not alter mitochondrial function in our study, it is likely that prolonged alteration of the plasma metabolome and/or cytokine levels is needed to observe changes in PBMC mitochondrial function. Previously, metabolic markers in PBMCs have been shown to respond to alterations in dietary intake (60, 61) and body weight (62). Our study demonstrates that PBMCs can also reflect differences in aerobic fitness level in healthy adult females, which further increases the potential of PBMCs as a biomarker to study the impact of lifestyle factors on human health.

Our study also demonstrated that a single, recent bout of exercise did not affect mitochondrial nor glycolytic metabolism in PBMCs from high-fit or low-fit females. However, a single bout of exercise was shown to increase mitochondrial PBMC metabolism in a previous study (25). Differences in sampling time might underlie these different observations, as Liepinsh et al. (25) sampled directly after exercise while we sampled 21 h after exercise. Since PBMC subsets are metabolically different (17, 18) and substantial shifts in PBMC subsets have been described after acute exercise (27–29), the increased mitochondrial PBMC function as observed by Liepinsh et al. could possibly be related to large differences in analyzed PBMC subsets between the two timepoints, whereas in our study PBMC composition was similar at baseline and after the recent bout of exercise. Second, differences in the metabolic substrates provided to the PBMCs during metabolic analysis might play a role. Fatty acid-dependent mitochondrial respiration was increased in permeabilized PBMCs after acute exercise (25), yet our study did not focus on substrate-dependent PBMC metabolism. Interestingly, in skeletal muscle, an acute, single bout of aerobic exercise is associated with alterations in metabolic fuel utilization, whereas regular bouts of aerobic exercise are associated with skeletal muscle adaptations, such as an increased number and function of mitochondria (63). This indicates that the impact of short- and longer-term exercise on cellular energy metabolism could be substantially different, and that differences between baseline and postexercise PBMC metabolism in our study could possibly have been detected when using different levels or combinations of metabolic substrates.

For comprehensive analysis of PBMC metabolism, we analyzed PBMC composition using flow cytometry in addition to our XF assays and showed that the major contributing PBMC cell types (∼90%) were not significantly different between high-fit and low-fit female individuals. This is in line with findings from previous studies (28, 64–67), although some studies also have shown that some lymphocyte subsets (e.g., CD3^+^, CD56^+^) were lower (68, 69) or higher (69) in high-fit compared with low-fit individuals. We also demonstrated that high-fit females had significantly higher monocyte (CD14^+^) and activated T cell (CD3^+^CD4^+^CD25^+^) frequencies as compared with low-fit females. However, we consider it unlikely that these differences impact our metabolic findings, because glycolytic PBMC function was not significantly different between the two groups, whereas monocytes and activated T cells were shown to rely more on glycolysis than the other PBMC subsets that we analyzed (17, 70). Furthermore, the contribution of the monocyte and activated T cell subset to the total PBMC pool is only ∼10% and the difference between the high-fit and low-fit group was only ∼2.4% of total, whereas respiration differences for the total population were around the magnitude of 20%. The overall stability in PBMC composition does not only temper the concerns for the influence of PBMC subset variability on PBMC biomarker responses, it also enforces the notion of PBMCs as a relevant biomarker tissue to examine lifestyle interventions. Importantly, studies that deal with major shifts in PBMC cell types, such as studies that include acute exercise interventions or disease pathology, might experience confounding by PBMC heterogeneity. Our study also included some limitations, such as the fact that we did not evaluate the effect of nutritional status on PBMC metabolism. For example, vitamin D status has been linked to mitochondrial PBMC function (61, 71). As we measured all our subjects in late autumn and winter, vitamin D intake from food becomes more important compared with summer. However, dietary vitamin D intake and status was not controlled for and could thus have acted as a potential confounder in our study.

In conclusion, using XF analysis of PBMC metabolism we showed that PBMCs from high-fit female individuals had increased mitochondrial function, which was not explained by changes in PBMC subsets, but instead implies an inherent higher oxygen consumption. Our study reveals a link between PBMC metabolism and levels of aerobic fitness, increasing the relevance of PBMC metabolism as a marker to study the impact of lifestyle factors on human health in future clinical and biological studies.

## SUPPLEMENTAL DATA

10.6084/m9.figshare.17111537 Supplemental Figure S1: https://doi.org/10.6084/m9.figshare.17111537.

10.6084/m9.figshare.16817437 Supplemental Figure S2: https://doi.org/10.6084/m9.figshare.16817437.

10.6084/m9.figshare.17125031 Supplemental Table S1: https://doi.org/10.6084/m9.figshare.17125031.

## GRANTS

This work was supported by NWO-WIAS Graduate Program Grant 2016 and the H2020-EU 3.2.2.1/2 PREVENTOMICS GA 818318 Grant.

## DISCLOSURES

No conflicts of interest, financial or otherwise, are declared by the authors.

## AUTHOR CONTRIBUTIONS

J.J.E.J., B.L., A.G.N., J.K., and V.C.J.d.B. conceived and designed research; J.J.E.J. and M.P. performed experiments; J.J.E.J. and M.P. analyzed data; J.J.E.J., M.P., J.K., and V.C.J.d.B. interpreted results of experiments; J.J.E.J. prepared figures; J.J.E.J. drafted manuscript; J.J.E.J., B.L., M.P., A.G.N., H.F.J.S., R.J.J.v.N., J.K., and V.C.J.d.B. edited and revised manuscript; J.J.E.J., B.L., M.P., A.G.N., H.F.J.S., R.J.J.v.N., J.K., and V.C.J.d.B. approved final version of manuscript.
